# Atrazine Induces Immunosuppression by Directly Targeting Lysozyme: Molecular Insights from Cellular and Multi-Spectroscopic Approaches in Eisenia Fetida

**DOI:** 10.3390/toxics14090825

**Published:** 2026-09-17

**Authors:** Penghang Ni, Xia Yuan, Shuqi Guo, Shaoyang Hu, Xiangxiang Li, Hengyu Song, Guang Tian, Xingchen Zhao, Wansong Zong, Jixin Su, Rutao Liu

**Affiliations:** 1 School of Environmental Science and Engineering, Shandong University, China-America CRC for Environment & Health, 72# Jimo Binhai Road, Qingdao 266237, China; 2 Shandong Soil Pollution Prevention and Control Center, 12#, Zhijinshi Street, Tianqiao District, Jinan 250012, China; 3 College of Geography and Environment, Shandong Normal University, 88# East Wenhua Road, Jinan 250014, China

**Keywords:** atrazine, lysozyme, multi-spectroscopy

## Abstract

Atrazine, a widely distributed and persistent triazine herbicide in the environment, has long raised ecological concerns. Existing research has mainly focused on its endocrine-disrupting effects, while immunotoxicity is a growing but relatively understudied area in atrazine research. By integrating cellular assays, multi-spectroscopy analysis, isothermal titration calorimetry (ITC), and molecular docking, this study reveals a molecular mechanism of atrazine-induced immunosuppression: Atrazine can directly target and inhibit the innate immune protein lysozyme, thereby triggering immunosuppression. In earthworm coelomocytes, atrazine exposure leads to decreased cell viability, loss of lysosomal membrane stability, and increased intracellular lysozyme activity. Further exploration of the molecular mechanism of this impairment reveals that atrazine spontaneously binds in the active site of lysozyme, driven mainly by hydrogen bonds and van der Waals forces. This binding causes the unfolding of the lysozyme protein backbone, rearrangement of the secondary structure, and changes in the microenvironment near tryptophan (Trp), thereby distorting the catalytic conformation, and sterically hindering substrate entry, ultimately resulting in the loss of its original catalytic activity. Collectively, this work demonstrates that atrazine can induce immunosuppression by directly acting on immune proteins, providing a novel mechanistic basis for the immunotoxicity and risk assessment of this herbicide.

## 1. Introduction

Atrazine, a synthetic triazine herbicide, is extensively employed for weed control in agricultural systems worldwide [1]. Since its introduction in the 1960s, atrazine has ranked second among the most widely used pesticides globally, owing to its cost-effectiveness and potent herbicidal activity [2]. However, atrazine possesses a relatively long half-life ranging from 41 to 231 days [3], coupled with high environmental mobility, leading to its frequent detection in surface water and groundwater, which raises significant ecological and public health concerns [4,5]. Prolonged and intensive application has resulted in atrazine residues in farmland soils reaching concentrations between 1.86 and 1100 mg·kg^−1^ in Chinese farmland soil, far exceeding regulatory limits (1.0 mg·kg^−1^) [6]. For instance, previous data have indicated that in the Qiqihar region of China, atrazine was continuously used for 6 years, resulting in soil accumulation of up to 167 mg·kg^−1^ [7]. Such widespread contamination not only threatens ecosystem integrity but also poses direct risks to non-target organisms, including crops, aquatic species, and humans.

Atrazine can disrupt the chromosome structure of cells and estrogen metabolism [8], and exhibits carcinogenic and endocrine-disrupting potential even at relatively low concentrations [9]. Besides the widely studied endocrine disruption and carcinogenic effects, the immune system, as the first line of defense against exogenous pollutants in the body, has gradually become a current research hotspot. The immune system’s dysfunction can trigger a series of ecological risks. For instance, the immune dysfunction of key soil organisms may lead to an imbalance in the soil microbial community, ultimately threatening the stability of the ecosystem. Existing studies have proved that atrazine can induce immune dysfunction [10,11]. However, a key question remains unresolved: Is this immunosuppression an indirect effect of cell damage, or does atrazine directly act on immune effector molecules through specific molecular interactions?

To address this question, this study selected the earthworms (Eisenia fetida) and the immune protein lysozyme as the research subjects. Within soil ecosystems, earthworms are important soil macroinvertebrates and ecosystem engineers, frequently employed as bioindicators of environmental pollution [12]. The coelomocytes of earthworms, as the primary defense mechanism of the innate immunity, protecting the organism against invading pathogens and xenobiotics [13]. At the same time, as a key defense protein, lysozyme also plays a crucial role in it. Functionally, lysozyme is an antibacterial, anti-inflammatory and immunomodulatory defense protein [14], which triggers bacterial cell lysis by hydrolyzing the glycosidic bond [15]. It also exhibits intrinsic antioxidant activity, further supporting its role in host defense [16,17]. Notably, the catalytic activity of lysozyme is strictly dependent on the precise spatial positioning of two key active site residues, glutamate (Glu) 35 and aspartate (Asp) 52 [18,19]. Additionally, Trp 62, Trp 63, and Trp 108 are located near the active site and stabilize the enzyme’s conformation through steric interactions [20,21]. This exquisite structure-function relationship makes lysozyme an ideal molecule for investigating whether pollutants can directly act on immune proteins.

In order to investigate the direct molecular interaction between atrazine and immune effector proteins, this study adopts earthworms and lysozyme as experimental subjects to determine whether the immunosuppression caused by atrazine is due to indirect cell damage or direct inhibition of immune biomolecules. This study mainly includes: (i) determination of atrazine-induced cytotoxicity, lysosomal membrane stability, and intracellular lysozyme activity changes in earthworm coelomocytes; (ii) multi-spectroscopic characterization of enzyme inhibition and conformational perturbation caused by atrazine; (iii) analysis of the binding thermodynamics and binding mode of atrazine with lysozyme. Linking cellular immune dysfunction to its underlying molecular mechanism can provide a novel foundation for the immunotoxicity risk assessment of atrazine.

## 2. Materials and Methods

### 2.1. Chemicals and Reagents

Atrazine (A821828, AR, 97%) was supplied by McLean Biochemical Technology Co., Ltd. (Shanghai, China). The lysozyme derived from egg white (which has a high structural homology with the lysozyme of invertebrates and is a widely accepted model protein) was purchased from Beijing Sun Biotechnology Co., Ltd. (Beijing, China). Roswell Park Memorial Institute (RPMI) 1640, Fetal bovine serum, penicillin/streptomycin, and were purchased from Gibco (Waltham, MA, USA). Cell Count Kit-8 (CCK-8) assay kit, Lysozyme assay kit, and Coomassie Brilliant Blue (CBB) protein assay kit were purchased from Nanjing Jiancheng Biochemical Corporation (Nanjing, China). phosphate-buffered saline (PBS, 0.2 mol/L, pH = 7.4, composed of 8 g/L NaCl, 0.2 g/L KCl, 1.44 g/L Na_2_HPO_4_, and 0.24 g/L KH_2_PO_4_) were purchased from Thermo Fisher Scientific Inc., Waltham, MA, USA.

### 2.2. Cellular Toxicity Assays

#### 2.2.1. Test Animals

Earthworms (E.fetida) were obtained from a professional breeding center in Qingdao, Shandong Province, China. They were raised in incubators to ensure that a constant temperature and humidity could be artificially maintained during their growth process, and they were regularly fed with cow dung. For the experiments, healthy, mature earthworms weighing between 300 and 600 mg, each with a clearly visible clitellum, were selected. To minimize inter-individual variability, coelomocytes were pooled from multiple earthworms per experimental group.

#### 2.2.2. Extraction and Culture of Coelomocytes

After the earthworms have evacuated their gut contents, wash them thoroughly and extract coelomocytes using a pre-prepared cell extraction solution [22]. Centrifuge the extracted cells at 4 °C and 3000 rpm for 5 min, then discard the supernatant. Wash the cell pellet twice with physiological saline. After washing, the cells were resuspended in the prepared culture medium and the cell density was diluted to 1 × 10^6^ cells/mL. Transfer the cell suspension into 96 or 24-well plates along with gradient-diluted atrazine solutions, and incubate in a sterile, CO_2_-free incubator at 25.0 ± 1 °C for 24 h. The final ethanol concentration was maintained below 0.1% (*v*/*v*). Preliminary experiments using an equivalent concentration of ethanol showed that there was no significant effect on the measured cell response under the current experimental conditions. The detailed operation steps can be found in Text S2.

#### 2.2.3. Cytotoxicity Assay

The cytotoxicity of atrazine to earthworm coelomocytes was assessed using the CCK-8 assay. In this assay, WST-8 is reduced by dehydrogenases within mitochondria in the presence of an electron mediator to produce an orange-yellow, water-soluble formazan product [23]. The intensity of the resulting color is inversely proportional to the level of cytotoxicity. After 24 h of cell culture in 96-well plates, an appropriate volume of CCK-8 reagent was added. After thoroughly mixing the system, it was placed back into the cell culture box and incubated at 25 °C for 2–4 h to allow the reaction system to fully develop color. Then, the absorbance of the reaction system at 450 nm was measured using a multi-mode microplate reader (Spark, TECAN, Männedorf, Switzerland). Cell vitality is calculated and assessed according to the following formula:

(1) $$\text{Cell}\;\text{viability}\;\left(\%\right)\;=\;\frac{{OD}_{a}-{OD}_{b}}{{OD}_{c}-{OD}_{d}}\;\times \;100\%$$

where *OD_a_* represents the absorbance of the experimental group (coelomocytes suspension + atrazine + CCK-8 reagent), *OD_b_* the absorbance of the experimental reference group (culture medium + atrazine + CCK-8 reagent), *OD_c_* the absorbance of the blank control group, and *OD_d_* the absorbance of the blank reference group. The results are expressed as a percentage, with the viability of cells in the blank control group (unexposed to the pollutant) defined as 100%.

#### 2.2.4. Measurement of Lysosome Membrane Stability

The stability of the lysosomal membrane was determined by the retention time of neutral red (NRRT). The coelomocytes were washed with PBS, and 20 μL of the cell suspension was mixed evenly with 80 μL of Ringer’s solution. Then, an appropriate amount of the mixed solution was dropped onto a glass slide, stained with the working solution of neutral red, and covered with a cover slip. The staining condition was observed under a microscope until 50% of the cells were stained. The detailed operation steps can be found in Text S3.

#### 2.2.5. Lysozyme Activity Assay

In this part of the experiment, we measured the changes in lysozyme activity both in the coelomocytes after atrazine exposure and in the enzyme directly exposed at the molecular level. For the cellular-level experiment, coelomocytes cultured for 24 h were ultrasonically disrupted in an ice-water bath to release intracellular contents, followed by detection according to the kit instructions. In a turbid bacterial suspension of a certain concentration, lysozyme lyses the bacteria, thereby reducing the turbidity and increasing the light transmittance. Thus, lysozyme activity can be determined based on the change in transmittance measured using a UV-Vis spectrophotometer (UV-2600, Shimadzu, Kyoto, Japan) at 530 nm. The relative activity of lysozyme was calculated as follows:

(2) $$\text{Lysozyme relative activity}\;=\;\frac{{\Delta T}_{1}}{{\Delta T}_{2}}\;\times {\;C}_{standard\;}\;\times \;N\;$$

where Δ*T_1_* and Δ*T_2_* are the transmittance difference in the measured sample at 15 s and 135 s, and the transmittance difference in the standard sample, respectively. *C_standard_* is the standard concentration. *N* is the dilution factor. The lysozyme activity of the blank control without atrazine was defined as 100%.

### 2.3. The Reaction System of Atrazine and Lysozyme

Before each experiment, the stock solution of lysozyme was prepared in deionized water. Atrazine was dissolved in an appropriate volume of anhydrous ethanol and then diluted with deionized water to prepare a 50 mg⋅L^−1^ solution. The final ethanol concentration in all reaction systems was maintained at ≤0.1% (*v*/*v*), which is below the threshold known to affect protein conformation or enzyme activity [24]. The reaction systems were prepared in 10 mL tubes by sequentially adding different volumes of atrazine stock solution, a fixed volume of PBS, and lysozyme stock solution. Finally, make up the volume to 10 mL using deionized water. All prepared systems were incubated at 25 °C for 30 min before experiments. To make the subsequent explanations clearer, the atrazine concentration of 0~5 mg⋅L^−1^ is considered the low-concentration, while the atrazine concentration of 10~50 mg⋅L^−1^ is regarded as the high-concentration. The upper limit of exposure concentration (40 mg·L^−1^) was determined based on environmental relevant levels: when the residual amount of atrazine in the soil reached 167 mg·kg^−1^, the estimated equilibrium concentration in the soil pore water was approximately 40 mg·L^−1^ (for detailed calculations, see Text S1).

### 2.4. Multi-Spectroscopy Analysis

#### 2.4.1. UV–Vis Absorption Spectra

The UV-vis absorption spectra of the atrazine–lysozyme system were measured using the UV-2600 spectrophotometer (Shimadzu, Kyoto, Japan). The control group used deionized water instead of lysozyme. For determination, the scanning wavelength range was 190 to 450 nm.

#### 2.4.2. Circular Dichroism (CD) Spectra

The CD spectra of lysozyme were determined using the J-1500 CD spectrometer (JASCO, Tokyo, Japan). Measure using a 1 mm path length quartz cell under the condition of nitrogen as the protective gas. The spectra were scanned from 190 to 260 nm at 200 nm·min^−1^, averaged over three accumulations, and corrected by subtracting the PBS baseline.

#### 2.4.3. Fluorescence Spectra Analysis

All fluorescence measurements were performed on the F-4600 fluorescence spectrometer (Hitachi, Tokyo, Japan). For determination, the photomultiplier tube was set at 750 V. The wavelength settings for the three fluorescence measurements are as follows. Fluorescence emission spectra: excitation wavelength (*λ_ex_*) = 290 nm, emission wavelength (*λ_em_*) = 290~450 nm. Synchronous fluorescence spectra: Δ*λ* (*λ_em_* − *λ_ex_*) was fixed at 15 nm (for Tyr) and 60 nm (for Trp), with *λ_ex_* scanned from 250 to 350 nm. 3D fluorescence spectra: *λ_ex_* = 200~280 nm, *λ_em_* = 260~420 nm. It must be noted that there is an internal filtering effect (IFE) in the reaction system, which will distort the measured data, so the influence of IFE should be corrected. We used the following formula to correct the obtained results [25]:

(3) $$F_{cor}\;=\;F_{obs}\;\times \;10^{\frac{\left(A_{ex}\;+\;A_{em}\right)}{2}}$$

where *F_cor_* is the fluorescence value after removing the IFE, and *F_obs_* is the measured fluorescence value. *A_ex_* and *A_em_* are the absorption values at the same wavelength in the UV-vis spectra experiment, respectively.

### 2.5. Isothermal Titration Calorimetry Investigation

Binding thermodynamics were investigated using the Malvern MicroCal PEAQ-ITC (Malvern Instruments Ltd., Malvern, UK). Before the start of titration, we used 0.02 M PBS as a solvent to configure various concentrations of lysozyme solution and atrazine solution, and filtered the configured solution before the experiment. During titration, we loaded lysozyme into the titration needle of the calorimeter and atrazine into the titration cell of the calorimeter. At a temperature of 298.15 K, a total of 13 injections (first 0.4 µL, followed by 12 injections of 3.0 µL each) were performed with a 120 s interval between injections and constant stirring at 750 rpm. A control titration (PBS added to atrazine) was subtracted from the raw data. The equation of Gibbs free energy change (ΔG) is:

(4) ΔG = ΔH−TΔS = RTlnK

where T is the thermodynamic temperature, and R is the gas constant [26].

### 2.6. Construction of the Action Model

Molecular docking simulations were performed using MOE (Chemical Computing Group Inc., Montreal, QC, Canada) to predict the docking site and mechanism of atrazine binding to lysozyme. The lysozyme (PDB ID: 2LYZ) was obtained from the RCSB PDB (https://www.rcsb.org/). Atrazine was obtained from Molview (https://molview.org/). Before the start of docking, lysozyme was dehydrated, and then polar hydrogen was added, followed by energy minimization and protonation of the lysozyme molecules. Then, the addressing module is used to select the appropriate docking site. After the selected site is docked, multiple docking results are obtained. We selected the structure that is most likely to form a combination from all the results for our study.

### 2.7. Statistical Analysis

The results obtained from the experiments are presented as the mean ± standard error (SEM, *n* = 3). Statistical significance among groups was determined by one-way analysis of variance (ANOVA) followed by Tukey’s post hoc test. Differences are considered statistically significant at *p* < 0.05.

## 3. Results and Discussions

### 3.1. The Toxicity of Atrazine to the Coelomocytes and the Intracellular Lysozyme of Earthworms

#### 3.1.1. Atrazine Exposure Resulted in Decreased Survival of the Coelomocytes

**Figure 1:**
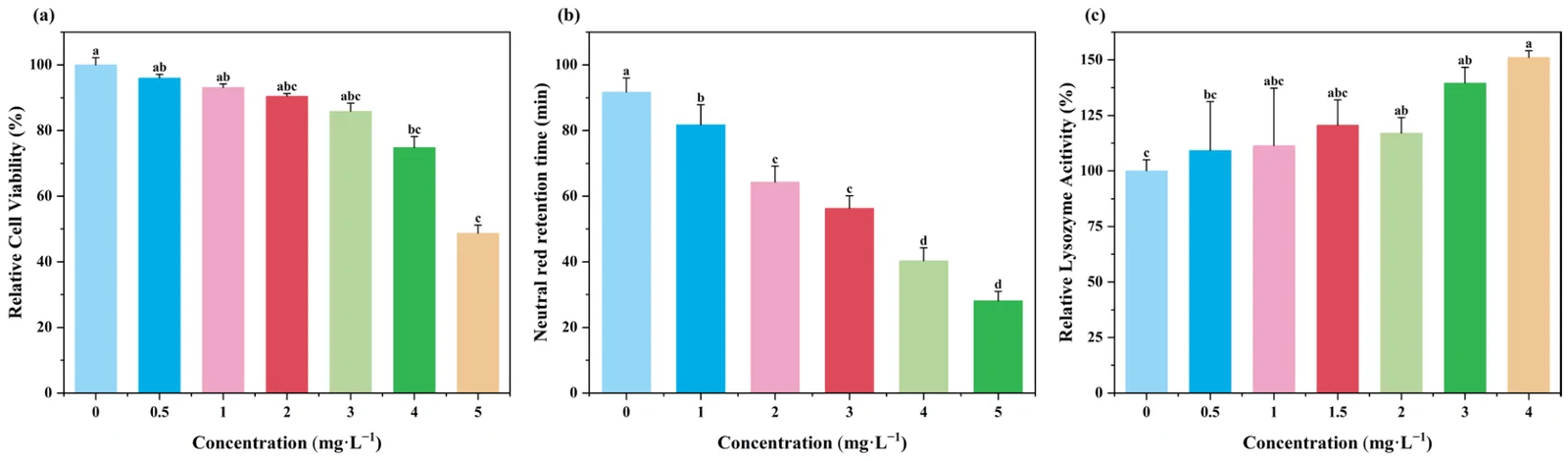
The exposure to atrazine led to changes in the cell vitality (**a**), NRRT (**b**), and intracellular lysozyme activity (**c**) of the earthworm coelomocytes. Different lowercase letters (a, b, c, etc.) above the columns indicate significant differences between groups (*p* < 0.05). **Experimental conditions**: c (atrazine) = 0, 0.5, 1, 1.5, 2, 3, 4, 5 mg⋅L^−1^.

To evaluate the immunotoxicity of atrazine at the cellular level, the effects of atrazine on the viability of earthworm coelomocytes and intracellular lysozyme activities were first examined. As shown in Figure 1a, the viability of the coelomocytes of earthworms decreased dose-dependently with the increase in atrazine concentration. Compared with the control group, when the concentration of atrazine reached 5 mg·L^−1^, the cell viability was significantly reduced to 47.46% (*p* < 0.05), indicating that atrazine had obvious cytotoxicity on earthworm immune cells.

#### 3.1.2. The Alteration of Lysosomal Membrane Stability Caused by Atrazine

To further explore the toxicity mechanism of atrazine, the retention time of neutral red was determined to reflect the stability of lysosomal membranes. Neutral red can be taken up and concentrated by lysosomes with normal physiological functions, so its retention time can be used to observe the permeability of lysosomal membranes [27]. Lysosomes are key cellular organelles in the immune response process. They can participate in antigen processing, inflammation regulation, and autophagy, among other processes, and are also important for the storage, maturation, and secretion of immune effector molecules such as lysozyme [28]. Studies have already demonstrated that increased lysosomal membrane permeability is a trigger for cellular dysfunction and even cell death [29].

As shown in Figure 1b, the retention time of neutral red in cells shows a downward trend (the NRRT time decreased from 91.71 min in the control group to 28.08 min at an atrazine concentration of 5 mg⋅L^−1^, representing a reduction of 30%), indicating that atrazine exposure increases the permeability of lysosomal membranes and disrupts the integrity of lysosomes. The loss of membrane stability may lead to the leakage of hydrolases from lysosomes into the cytoplasm, triggering apoptosis or necrosis, and thereby causing a decline in cell viability.

#### 3.1.3. Atrazine Exposure Resulted in Changes in Intracellular Lysozyme Activity

Figure 1c shows the activity of lysozyme in the coelomocytes of earthworms after exposure to atrazine. Notably, the intracellular lysozyme activity of earthworm coelomocyte was significantly increased when exposed to low concentrations of atrazine compared with the control. This phenomenon indicates that when coelomocytes detect the invasion of atrazine, they may trigger their own stress defense response [30]. Through upregulation of lysozyme expression or activation of related regulatory pathways, they enhance their own immune defense against external stress to resist the invasion of atrazine [31]. For instance, exposure of the roundworm Caenorhabditis elegans showed a clear hormesis dose–response to irradiation when subjected to different concentrations of herbicides [32].

**Figure 2:**
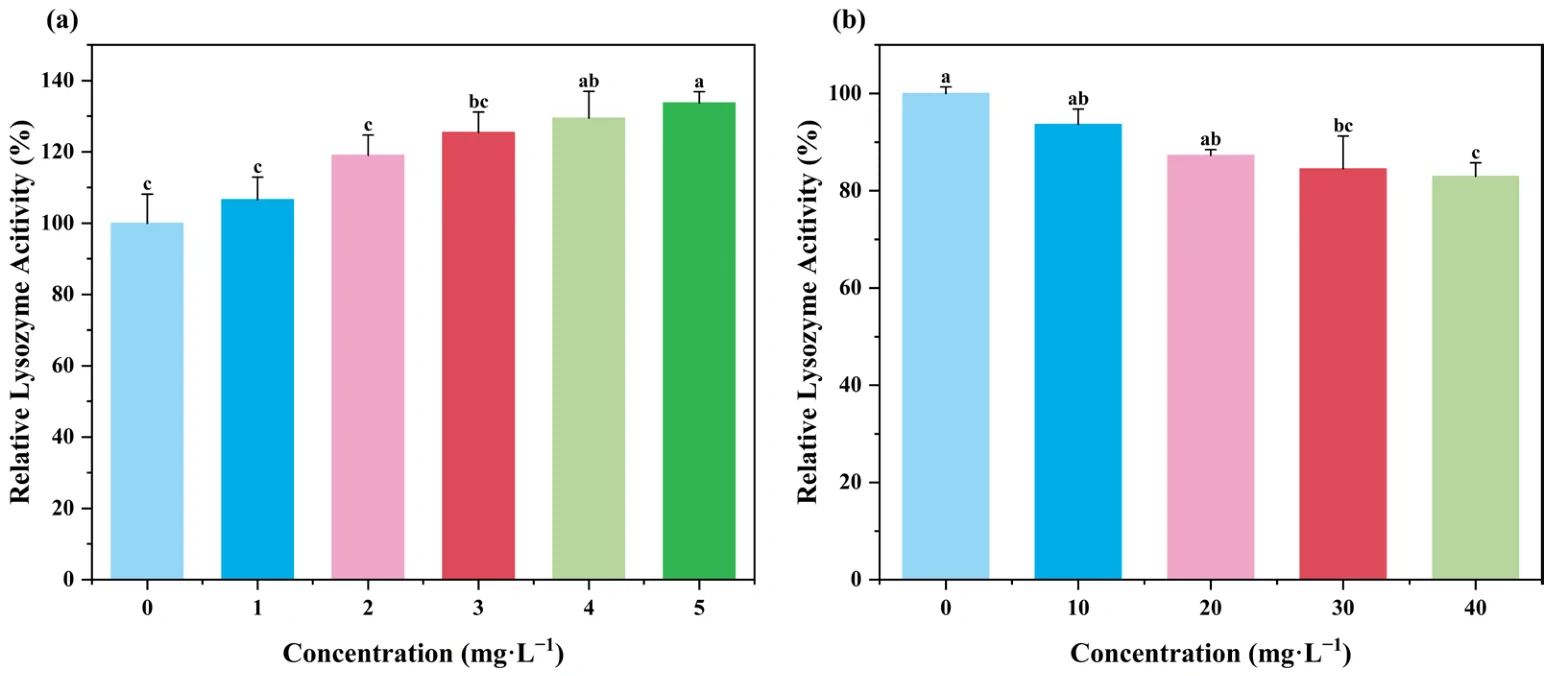
The changes in extracellular relative lysozyme activity after exposure to low-concentration (**a**) and high-concentration (**b**) atrazine. Different lowercase letters (a, b, c.) above the columns indicate significant differences between groups (*p* < 0.05). **Experimental conditions**: c (lysozyme) = 2 μM, c (atrazine) = 0, 1, 2, 3, 4, 5, 10, 20, 30, 40 mg⋅L^−1^.

Furthermore, by further integrating the experimental results at the cellular level, atrazine disrupts the stability of the lysosomal membrane, leading to the abnormal release of intracellular lysozymes. However, it should be noted that merely relying on activity detection is insufficient to distinguish between an increase in enzyme quantity (expression level) and an improvement in the catalytic efficiency (enzyme activation) within the enzyme. Therefore, the increase in intracellular lysozyme activity may be caused by at least two mechanisms working together: (i) under low concentration stress conditions, cells compensate by enhancing the expression and secretion of lysozyme; and (ii) atrazine directly affects the conformation of the lysozyme molecule, thereby altering its catalytic properties. To distinguish between these two possibilities, we conducted parallel molecular-level experiments using purified lysozyme (Figure 2a), and the results will be discussed in the following text.

### 3.2. The Direct Influence of Atrazine on the Catalytic Action of Lysozyme

In order to investigate whether atrazine can directly act on lysozyme and affect its catalytic function, the changes in enzyme activity were measured at the molecular level. The results of the in vitro molecular analysis of the lysozyme are shown in Figure 2. Under low-concentration atrazine treatment, the activity of lysozyme increased (Figure 2a), demonstrating that atrazine can directly regulate the catalytic efficiency of lysozyme through molecular interactions and does not rely on cellular regulatory processes. This finding is complementary to the cellular-level observation in Figure 1c; although the increase in lysozyme activity in the cavity cells may reflect a compensatory response induced by stress, the molecular-level results in Figure 2a provide direct evidence that atrazine can also affect the inherent catalytic activity of this enzyme through conformational changes. However, the current experimental design is unable to distinguish between the enhancement of enzyme expression and the increase in specific activity. Future research can employ quantitative protein detection methods (Western blotting) and transcriptional analysis (qPCR) and other techniques to comprehensively elucidate the relative contributions of these two mechanisms at the cellular level.

At high concentrations (Figure 2b), as the atrazine concentration continued to increase, the catalytic activity of lysozyme gradually decreased. This confirmed that atrazine could directly weaken the antibacterial function of lysozyme. The catalytic activity of lysozyme is highly dependent on the precise spatial conformation of its active center glutamic acid 35 (Glu 35) and aspartic acid 52 (Asp 52) [33]. Given that protein structure is the basis of its function [34], we hypothesized that atrazine might affect its activity by disturbing the spatial structure of lysozyme. Subsequent multi-spectral analysis aims to provide direct structural evidence for this hypothesis.

### 3.3. The Structural Perturbation of Lysozyme Induced by Atrazine

#### 3.3.1. The Loosening of the Peptide Chain Backbone

**Figure 3:**
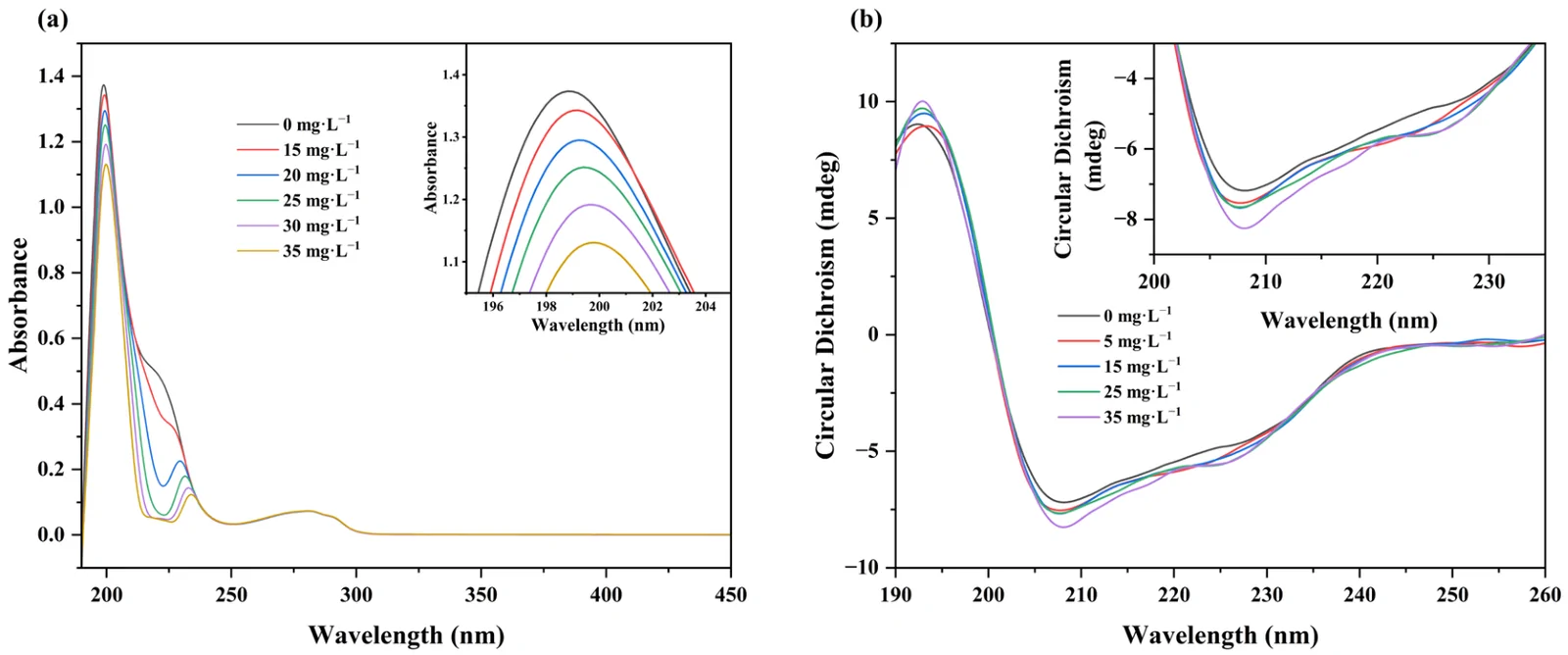
UV-vis absorption spectra (**a**) and CD spectra (**b**) of lysozyme after atrazine exposure. **Experimental conditions**: In the UV-vis absorption spectra, c (lysozyme) = 2 μM, c (atrazine) = 0, 15, 20, 25, 30, 35 mg⋅L^−1^. In the CD spectra, c (lysozyme) = 5 μM, c (atrazine) = 0, 5, 15, 25, 35 mg⋅L^−1^.

UV-vis absorption spectroscopy can be used to detect the differences in absorbance between different protein conformations, thereby determining whether the ligand causes a structural change in the protein [35]. Scanning both the sample (lysozyme + atrazine) and the corresponding blank (atrazine + buffer) allows us to correct for the contribution of atrazine [36]. In Figure 3a, absorption peaks near 197 nm and 280 nm can be observed. There is a distinct and strong absorption peak around 197 nm. This characteristic peak mainly originates from the π-π* electronic transition of the carbonyl group (C=O) on the peptide bond (-CO-NH-) in the protein polypeptide backbone. Its intensity and position are highly sensitive to the conformational state of the peptide chain [36,37]. Therefore, the change in this absorption peak is often used as an important spectral indicator for monitoring the folding or extension of the protein backbone. The change in the peak near 280 nm represents the change in the microenvironment near specific amino acids in the lysozyme [38], and this absorption peak is often used to monitor the microenvironment of specific amino acids.

In the low-concentration range (Figure S1), as the atrazine concentration increased, the absorption peak at 197 nm slightly enhanced, indicating that the atrazine molecules had begun to interact with the lysozyme, causing a local conformational adjustment of the lysozyme and altering its catalytic activity. When the concentration exceeded a certain threshold, as the atrazine concentration increased, the lysozyme skeleton absorption peak showed a gradual decrease and a slight red shift. This suggests that atrazine has damaged the structure of lysozyme, causing the framework of lysozyme to become loose and unfolded [39]. The red shift in the absorption peak indicates that the energy required for the transition of the peptide bond π-π* has decreased, and the polarity and hydrophilicity of the microenvironment around the peptide bond have increased [40]. The absorption peak at 280 nm shows no significant change, suggesting that the binding of atrazine may not have significantly altered the local microenvironment of specific amino acid residues [41]. However, the influence of atrazine on the overall conformation of lysozyme still needs to be further confirmed.

#### 3.3.2. Rearrangement of Secondary Structure and Partial Denaturation

CD spectra are a classic method for characterizing the secondary structure of proteins, where the signals of characteristic peaks are closely related to the electronic transitions of specific folding patterns [42]. In Figure 3b, the peak at 208 nm reflects the π-π* electron transition of the lysozyme α-Helix structure, while the peak at 220 nm reflects the π-π* electron transition of both the lysozyme α-Helix structure and the random coil structure [43,44]. The intensity and position changes in these two characteristic peaks are often used to infer the content and stability of ordered structures such as α-Helix in proteins. Based on the results obtained from CD spectroscopic measurements, the reaction between lysozyme and atrazine would enhance the intensity of the negative peak, indicating an increase in the α-Helix structure of lysozyme. The degree of structural change is positively correlated with the concentration of atrazine, indicating that this conformational perturbation has a significant concentration-dependent nature.

The data on the secondary structure composition of lysozyme is shown in Table S1. Previous studies have shown that the binding of organochlorine pesticide endosulfan and viologen herbicide to lysozyme does not lead to significant changes in the structure of lysozyme, and the integrity of its secondary structure remains relatively unchanged [45,46]. However, this study found that as the concentration of atrazine increased, the α-Helix and Turns of lysozyme increased, while the β-Sheet structure decreased. This rearrangement of secondary structures is a typical feature of protein misfolding and partial denaturation, which may be caused by electrostatic and hydrophobic interactions [37]. Moreover, the increase in α-Helix is often accompanied by protein elongation and expansion of the hydrogen bond environment, indicating that the structure of lysozyme has been expanded in all directions [47,48], which is consistent with the conclusion of loose backbone observed by UV-vis absorption spectroscopy.

#### 3.3.3. The Changes in the Microenvironment of the Fluorescence Chromophore

**Figure 4:**
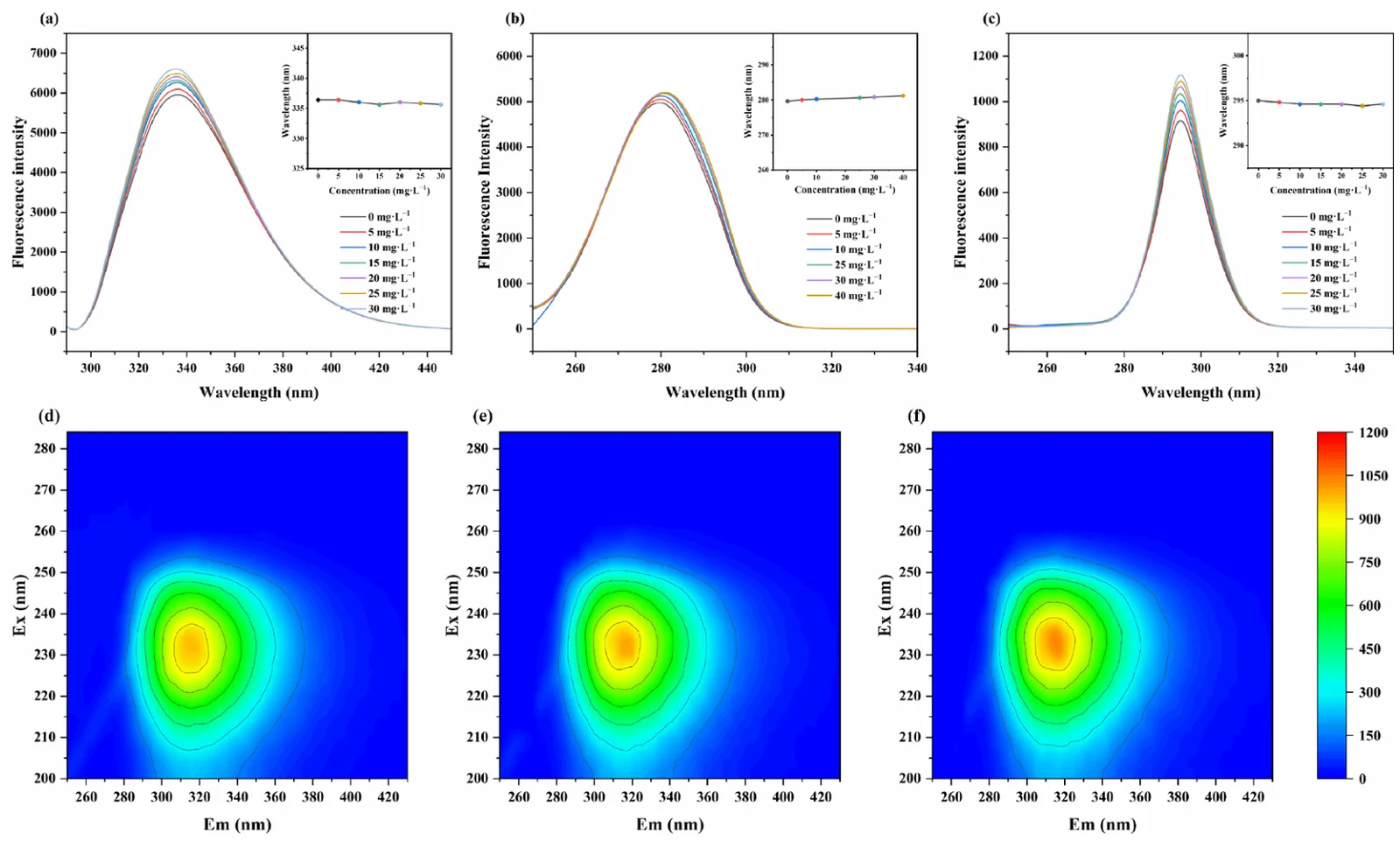
Fluorescence spectra and 3D fluorescence spectra of lysozyme after atrazine exposure. Fluorescence emission spectra of atrazine–lysozyme (**a**), synchronous fluorescence spectra with wavelength differences of 60 nm (**b**) and 15 nm (**c**). 3D fluorescence spectra of the atrazine–lysozyme system (**d**–**f**). **Experimental conditions**: c (lysozyme) = 2 μM, c (atrazine) = 0, 5, 10, 15, 20, 25, 30, 40 mg⋅L^−1^. Among them, the atrazine concentration in Figure (**d**–**f**) is 0, 10, 20 mg⋅L^−1^.

The fluorescent chromophores of a protein may also be affected due to changes in its own structure. Therefore, by analyzing the fluorescent chromophore, the mechanism of atrazine’s effect on lysozyme can be further studied [41]. Lysozyme fluorescence is derived from tryptophan, tyrosine, and phenylalanine residues, where tryptophan and tyrosine residues predominate and phenylalanine residues contribute less and are difficult to detect [21,36]. It should be noted that, based on the conclusion of the UV-vis spectra experiments, the IFE effect cannot be ignored [38]. The results after IFE correction are shown in Figure 4.

Figure 4a reveals the spectrogram of the fluorescence emission. The emission fluorescence peak of lysozyme appears around 336 nm. The form and location of the peak do not change with the variation in atrazine concentration, but the peak intensity gradually increases. This suggests that atrazine interacts with the fluorescent chromophore of lysozyme. The reason for this phenomenon might be that micelles were formed in the atrazine–lysozyme system [39]. The atrazine molecule has an organochlorine heterocyclic structure and is hydrophobic in itself. When bound to the lysozyme, it is likely to be close to or cover the chromophore of the lysozyme. This binding results in the displacement of water molecules around amino acid residues of lysozyme by atrazine or is more tightly wrapped by the protein skeleton, forming a more hydrophobic and more rigid local environment. As a result, the fluorescence quantum yield increases, the non-radiative transition pathways decrease, and ultimately, the peak intensity is enhanced [39,49].

The fluorescence emission spectra of proteins can reflect the overall changes in protein fluorescence, while the synchronous fluorescence spectra can observe whether the microenvironment of certain specific amino acids has changed [50,51]. Figure 4b,c show the synchronous fluorescence spectra of lysozyme under a wavelength difference of 60 nm and 15 nm. They respectively reflect the fluorescence intensity changes in Trp and Tyr, and both increase with the increase in atrazine concentration. However, the exposure of atrazine did not change the shape and position of the synchronous fluorescence peaks, proving that the microenvironments around these two amino acids did not undergo significant changes.

Overall, the fluorescence spectra of all three types showed an increasing trend of fluorescence signals, indicating that the interaction between atrazine and lysozyme affects the microenvironment of the fluorescent chromophore. This interaction encapsulates Trp and Tyr, resulting in a similar micellar-like enhancement effect, increasing the fluorescence quantum yield and reducing the energy loss from collisions [52], ultimately leading to an increasing trend in all the detected fluorescence signals.

#### 3.3.4. The Changes in the 3D Fluorescence Spectra of Lysozyme

In contrast to the fluorescence spectra shown in Figure 4a–c, which report the local microenvironment of specific residues, the 3D fluorescence spectra (Figure 4d–f) can simultaneously scan the excitation and emission wavelengths, thereby providing information on the overall conformational state of the protein and revealing the overall structural perturbations [36]. Together, they jointly demonstrate that atrazine can induce conformational changes in lysozyme at both the local and overall levels. The 3D fluorescence spectra of lysozyme were corrected, and the Rayleigh scattering peak was subtracted to obtain the spectral graph shown in Figure 4d–f. The characteristic peaks in the figure originate from the π-π* transition of aromatic amino acids, reflecting the fluorescence properties of lysozyme [53]. This result is consistent with the aforementioned fluorescence spectra analysis, jointly supporting the conclusion that atrazine can cause conformational changes in lysozyme and affect its fluorescent chromophore.

Furthermore, these results also provide a mechanistic explanation for the decrease in lysozyme activity. Multispectral experiments show that atrazine exposure will damage the structure of lysozyme. The conformational change caused by this binding may cause the spatial position of Glu 35 or Asp 52 in the active center to shift, affecting the precise matching with the substrate, thereby weakening the catalytic function of the enzyme.

### 3.4. Thermodynamic Studies of Lysozyme Binding to Atrazine

**Figure 5:**
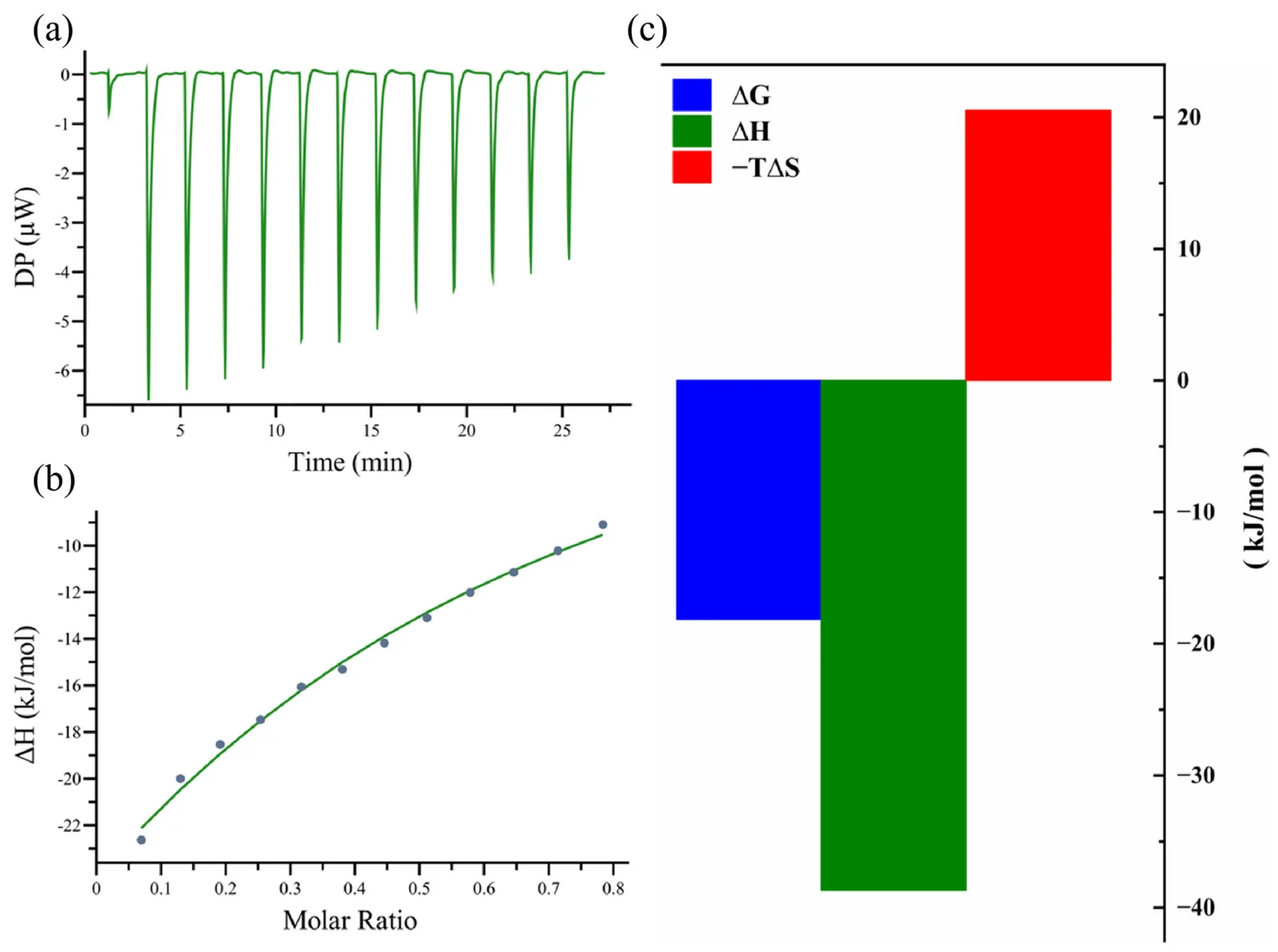
Panel of ITC results of atrazine binding to lysozyme. Raw thermal power signals from reverse titration of lysozyme into atrazine solution; negative peaks indicate exothermic binding (**a**). Integrated and baseline-corrected binding isotherm, showing the heat changes associated with each injection during the titration process (**b**). Bar chart showing the thermodynamic parameters ΔH, −TΔS, and ΔG, illustrating the relative enthalpy and entropy contributions to the binding free energy (**c**). **Experimental conditions**: c (lysozyme) = 800 μM, c (atrazine) = 200 μM.

Isothermal titration calorimetry can directly obtain the thermodynamic parameters of the reaction system, including enthalpy change (ΔH), entropy change (ΔS), binding constant (K_D_), and binding stoichiometry (n) [54]. Figure 5 presents the data obtained from the isothermal titration calorimetry. For titration, the lysozyme solution was dropped into the atrazine solution by reverse titration. The DP curve shows a negative peak, and the ΔH value of the reaction is also negative. This confirms that the binding of atrazine to lysozyme is an exothermic process. With titration of the lysozyme solution, atrazine in the titration pool was continuously consumed, and in combination with lysozyme, the peaks of the DP images gradually decreased and stabilized. The ΔG < 0 of the binding process, which means that the combination process proceeds spontaneously. In general, if the value of K_D_ is in the 10^−7^~10^−8^ range, it indicates that there is a strong binding interaction in the reaction. The K_D_ of atrazine binding to lysozyme is about 6.67 × 10^−4^ M, indicating a relatively weak binding affinity rather than a high-affinity interaction [55,56].

The ΔH < 0 of the reaction indicates that a non-covalent bond is formed between the two interacting molecules, and the reaction is mainly driven by the contribution of favorable enthalpies. The ΔS < 0 indicates that during the course of the reaction, the orderliness of the system increases. This might be because after binding, the conformation of the lysozyme tends to become fixed, and the rearrangement of solvent molecules limits the degrees of freedom of the system. This is consistent with the structural stiffening phenomenon observed in CD and fluorescence spectra. The −TΔS > 0 may be caused by the conformational change, the combined system has reduced rotation and translation degrees of freedom, and limited migration degrees of freedom [57]. By jointly analyzing ΔH and ΔS, the binding forces present in the reaction can be determined. In this study, the ΔH and ΔS of the binding of atrazine to lysozyme were both negative, indicating that the binding process is an exothermic and ordered process. This usually implies that the intermolecular forces existing in the binding process are mainly hydrogen bonds and van der Waals forces [58]. The reason is that the formation of hydrogen bonds usually involves strict orientation and distance requirements, which leads to a significant increase in system order (ΔS < 0) and the release of heat (ΔH < 0); while van der Waals forces, as a short-range attraction, their contribution is mainly reflected in the negative enthalpy change [59].

### 3.5. A Binding Model for the Atrazine–Lysozyme Complex

Molecular docking is an important technique that can be used to determine the possible binding mode between a protein and a ligand. Given the 3D structure of a small molecule and a protein, molecular docking software can fit the small molecule to the target of the protein, thereby predicting the binding structure and binding energy of the two [60]. Moreover, molecular docking can identify the energetically most favorable conformation of ligands within the active site of a protein. Then, the scoring function is used to rank these conformations [61]. In molecular docking simulations, the scoring function of the docking conformation usually takes the binding energy as the key evaluation criterion. Generally, the docking site with the lowest binding energy is considered to represent the thermodynamically most stable ligand–protein binding mode, and thus is most likely to reflect the actual interaction site. In this study, based on this principle, we selected the conformation with the lowest binding energy from all the generated docking conformations as the representative model. Based on the spatial position, amino acid residue composition, and interaction type of this optimal conformation, we further explained the possible binding mechanism and molecular recognition basis between atrazine and lysozyme.

**Figure 6:**
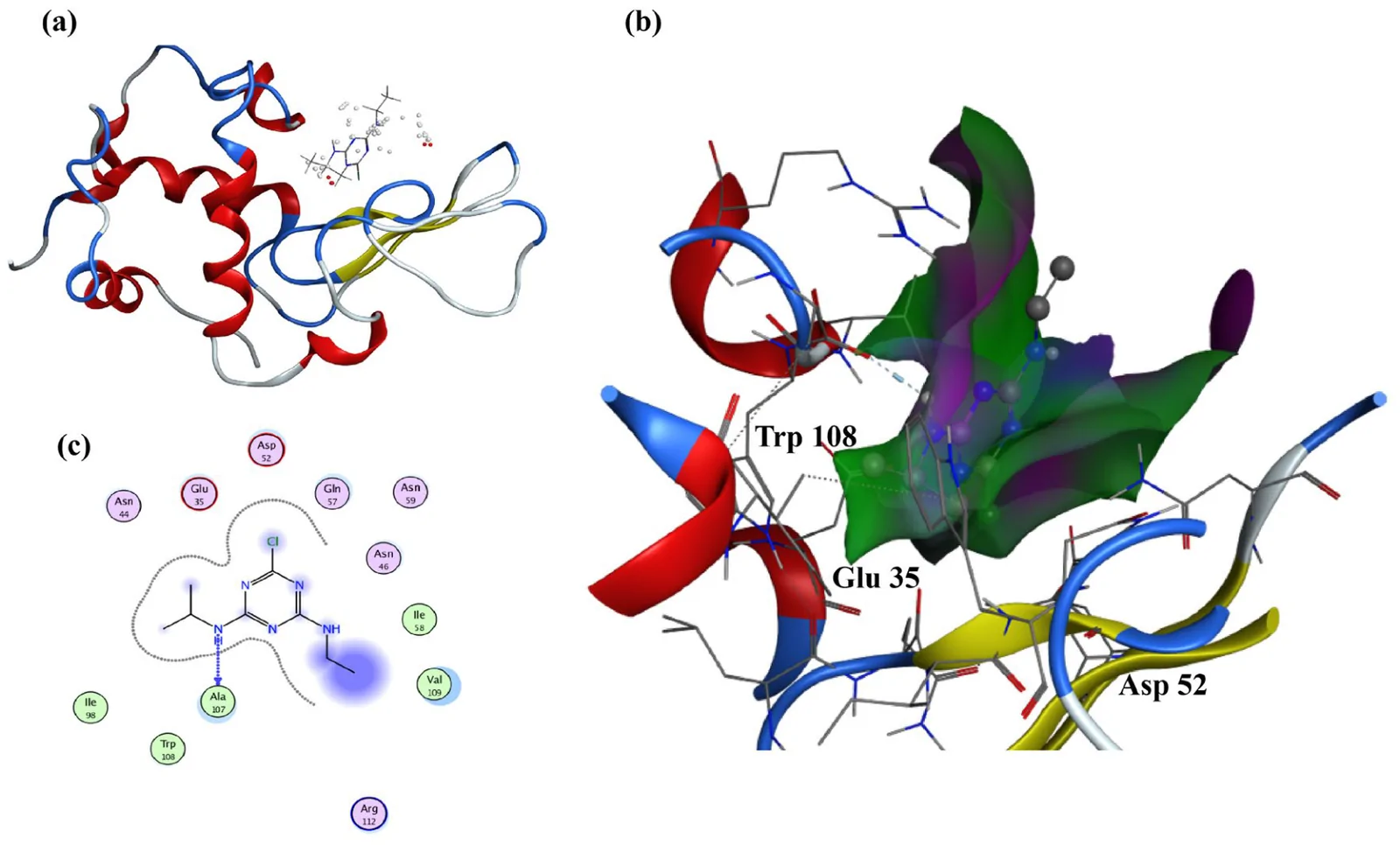
The action models of atrazine and lysozyme. Profile of binding positions and docking sites (**a**), Gaussian contact profile (**b**), and amino acid profile at the binding site (**c**).

Previous studies have shown that the α domains and β domains are the two important regions of lysozyme, and the active sites Glu 35 and Asp 52 of lysozyme are located in the gap of this domain, which occupies a critical position of the catalytic activity of lysozyme [33,62]. Figure 6a illustrates the docking sites of atrazine and lysozyme, and it can be seen that these sites are located in this large gap. Figure 6b is a Gaussian contact map showing the relative position and the possible binding force of lysozyme binding to atrazine. Different colors are used to represent different types of forces. Among them, the green force is the hydrophobic force, the pink force is the hydrogen bond force, and the blue force is the mild polar force. The result of the docking indicates that there may be hydrophobic force and hydrogen bond force in the binding between atrazine and lysozyme. This result is consistent with the conclusion obtained from the aforementioned ITC experiment. Figure 6c shows the amino acids present near the binding site. Among them, Trp 108 was found in the lysozyme binding pocket. It has been shown that among all the Trp in lysozyme, Trp 62 and Trp 108 are the most significant fluorophores [63]. This finding provides a plausible explanation for the changes observed in the fluorescence spectra upon atrazine exposure. In addition, Asp 52 and Glu 35 are both present in the binding pocket. The binding of atrazine may cause the positions of these two sites to change or to be occupied, preventing them from performing their normal functions, ultimately resulting in the inability of lysozyme to effectively combine with the substrate. This further explains why exposure to atrazine leads to a decrease in lysozyme activity and inhibition of its normal functions.

In summary, atrazine induces significant local conformational changes in the key region of the active center of lysozyme containing Trp 108, Asp 52, Glu 35, etc., by specifically binding to this region. This binding results in enhanced fluorescence of lysozyme. At the same time, the binding of atrazine occupies and distorts the catalytic core of the enzyme, obstructs the entry of substrates and the reaction, and destroys the conformation around key catalytic residues, leading to the inhibition of lysozyme activity.

## 4. Conclusions

This study, by integrating cellular and molecular approaches, demonstrated that atrazine can directly bind to the lysozyme of the innate immune protein and disrupt its structural integrity, thereby inducing immunosuppression. In coelomocytes of earthworms, the exposure of atrazine would reduce cell viability, destabilize the stability of the lysosome membrane, and cause abnormal elevation of the activity of intracellular lysozyme. By tracing the molecular mechanism of this functional disorder, it further proved that atrazine would spontaneously bind to the active site of lysozyme, with a K_D_ of approximately 6.67 × 10^−4^, reflecting a moderate binding strength. The binding of the two is mainly driven by hydrogen bonds and van der Waals forces, triggering the extension of the peptide chain, the rearrangement of secondary structure, and the formation of a hydrophobic microenvironment around the tryptophan residue. These conformational changes distorted the spatial positions of key catalytic residues (Glu 35 and Asp 52), hindered the binding of the enzyme to the substrate, and ultimately reduced the activity of lysozyme. When the exposure concentration of atrazine reached 40 mg⋅L^−1^, the activity of lysozyme was inhibited to 81.32% of the control group. These results link the immunotoxicity of atrazine to its direct induction of protein inactivation, providing a new perspective for the study of atrazine’s immunotoxicity. Finally, it should be noted that this study focused on the parent compound atrazine, while its major metabolites such as deethylatrazine (DEA) and deisopropylatrazine (DIA) were not examined. Given that transformation products may also exert biological effects, future investigations are warranted to evaluate their potential immunotoxicity and possible combined effects with atrazine.

## Data Availability

The original contributions presented in this study are included in the article/Supplementary Material. Further inquiries can be directed to the corresponding authors.

## Supplementary Materials

The following supporting information can be downloaded at https://www.mdpi.com/article/10.3390/toxics14090825/s1: Text S1: Estimating the potential water-phase atrazine concentration from soil residues; Text S2: Extraction and Cultivation of Coelomocytes of Earthworms; Text S3: The experimental process of the neutral red staining method; Figure S1: Changes in the UV-vis absorption spectroscopy of lysozyme under low-concentration atrazine treatment; Table S1: Secondary structure composition (%) of lysozyme after atrazine exposure. References [64,65,66,67,68,69] are cited in the supplementary materials.
