# Supplementary material for: Atrazine Induces Immunosuppression by Directly Targeting Lysozyme: Molecular Insights from Cellular and Multi-Spectroscopic Approaches in Eisenia Fetida
Source: Toxics. 2026 Sep 17;14(9):825. doi: 10.3390/toxics14090825 (PMC13611554; doi:10.3390/toxics14090825)
Supplement: Supplementary file 1 [file toxics-14-00825-s001.zip › toxics-4545098-supplementary.pdf]

Atrazine Induces Immunosuppression by Directly Targeting Lysozyme: Molecular Insights from Cellular and Multi-Spectroscopic Approaches in Eisenia Fetida

Penghang Ni [1], Xia Yuan [2], Shuqi Guo [1], Shaoyang Hu [1], Xiangxiang Li [1], Hengyu Song [1], Guang Tian [1], Xingchen Zhao [1], Wansong Zong [3], Jixin Su [1,*] and Rutao Liu [1,*]

[1] School of Environmental Science and Engineering, Shandong University, China-America CRC for Environment & Health, 72# Jimo Binhai Road, Qingdao 266237, China

[2] Shandong Soil Pollution Prevention and Control Center, 12#, Zhijinshi Street, Tianqiao District, Jinan 250012, China

[3] College of Geography and Environment, Shandong Normal University, 88# East Wenhua Road, Jinan 250014, China

* Correspondence: jxsu@sdu.edu.cn (J.S.); rutaoliu@sdu.edu.cn (R.L.); Tel./Fax: +86-0531-88365489 (R.L.)

Supporting Information

The listed captions

Text S1. Estimating the potential water-phase atrazine concentration from soil residues.

Text S2. Extraction and Cultivation of Coelomocytes of Earthworms.

Text S3. The experimental process of the neutral red staining method.

Figure S1. Changes in the UV-vis absorption spectroscopy of lysozyme under low-concentration atrazine treatment.

Table S1. Secondary structure composition (%) of lysozyme after atrazine exposure.

Text S1. Estimating the potential water-phase atrazine concentration from soil residues.

In the soil of the field, the residual concentration of atrazine is usually expressed as the mass per unit dry weight of the soil (mg/kg), while in the cell culture experiments of this study, the exposure concentration is expressed as the mass per unit volume of the culture medium (mg/L). Due to the different media, these two cannot be directly converted. To establish the scale correspondence between the field pollution level and the laboratory exposure concentration, this supplementary explanation adopts the soil-water partition equilibrium method to estimate the potential pore water concentration of atrazine in highly polluted farmland soil.

In the soil-water two-phase system, when adsorption-desorption reaches equilibrium, the distribution behavior of atrazine between the solid phase (soil particles) and the liquid phase (soil pore water) can be described by a linear distribution model [1, 2].

$$K_d = \frac{C_s}{C_w} \tag{S1}$$

In formula S1:

C_s: The concentration of atrazine adsorbed on the solid phase of the soil (mg/kg).

C_w: The concentration of atrazine dissolved in the soil pore water (mg/L).

The total amount of atrazine in one kilogram of dry soil is equal to the sum of the adsorbed amount on the solid phase and the dissolved amount in the liquid phase [3]:

$$C_T = C_s + \theta C_w \tag{S2}$$

In formula S2:

θ: Gravitational method soil moisture content (L/kg). The soil moisture content in the fields is generally between 0.15 and 0.30 L/kg.

The above formula is then simplified to obtain the estimation formula for the water phase concentration:

$$C_w = \frac{C_T}{K_d + \theta} \tag{S3}$$

In this study, a value of C_T = 167 mg/kg was selected as the representative value for the high pollution scenario. This figure was derived from the measured data of farmland soil in the Qiqihar area of China, where atrazine has been continuously applied for a long time [4]. The K_d value of atrazine in general soil is usually around 1 to 5 L/kg [5]. In the low organic carbon soil of Pakistan, the K_d value is 0.4 - 1.5, and in the two types of soil in Brazil, the K_d values are 2.6 and 2.1 [6, 7]. The calculation results show that under the condition where the atrazine residue in the soil is 167 mg/kg, the concentration of atrazine in the soil pore water at equilibrium can reach approximately 40 mg/L. This supports the selection of the upper limit of the in vitro concentration in concentration-response studies.

Text S2. Extraction and cultivation of coelomocytes of earthworms.

Select a sufficient number of appropriately sized adult earthworms and place them in culture dishes lined with moist filter paper. Cover the dishes and incubate in darkness for 24 hours to allow the earthworms to fully clear gut contents. After gut clearance, wash the earthworm surface with deionized water and physiological saline respectively. Once cleaned, extract coelomocytes using a pre-prepared extraction solution (containing 10 mg/mL guaifenesin, 2.5 mg/mL disodium EDTA, 5% ethanol, 95% physiological saline, pH=7.4). After extraction, centrifuge the cells at 4°C and 3000 rpm for 5 minutes, then discard the supernatant. Resuspend the pellet in physiological saline and centrifuge again. Repeat the washing step twice, then transfer the cells into culture medium (composed of 89% RPMI 1640 medium, 10% fetal bovine serum, and 1% penicillin/streptomycin). Seed the cells into 96-well or 24-well plates, add gradient concentrations of atrazine solution along with culture medium into each well, and incubate in a sterile, CO_2-free incubator at 25.0 ± 1 °C for 24 hours.

Text S3. The experimental process of the neutral red staining method.

The stability of lysosomal membranes was evaluated by the neutral red retention time (NRRT). The neutral red working solution was prepared by dissolving 20 mg of neutral red powder in 1 ml of dimethyl sulfoxide, and then adding 2.5 ml of physiological Ringer's solution (0.0426 g of Na_2HPO_4, 0.0544 g of KH_2PO_4, 0.2527 g of $NaHCO_3$, 0.2706 g of $MgSO_4$, 4.14 g of NaCl, 0.3549 g of KCl, and 0.4179 g of $CaCl_2$ dissolved in 1000 ml of deionized water). For the determination, the co-cultured earthworm coelomocytes with atrazine for 24 h were transferred to a centrifuge tube, centrifuged at 4°C and 3000 rpm for 5 minutes, and the supernatant was discarded. The remaining coelomocytes were resuspended and washed with PBS twice. 20 μL of the washed coelomocytes supernatant was mixed with 80 μL of physiological Ringer's solution. 20 μL of the mixture was dropped onto a clean slide, and 20 μL of the neutral red working solution was added for staining. Then a cover slip was placed on top. The staining of the cells was observed under an optical microscope at 400x magnification every 2 minutes until 50% of the cells were stained. The time taken for staining was recorded as the NRRT.

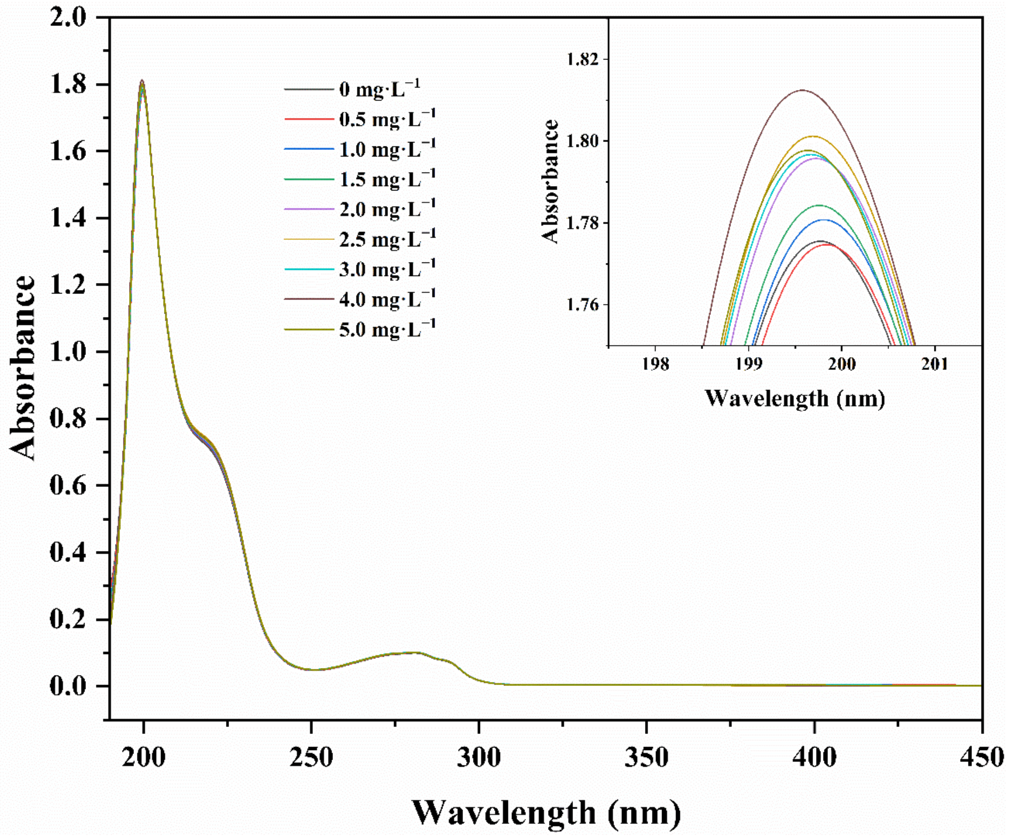

Figure S1. Changes in the UV-vis absorption spectroscopy of lysozyme under low-concentration atrazine treatment.

Table S1. Secondary structure composition (%) of lysozyme after atrazine exposure.

Atrazine Concentration ($mg \cdot L^{-1}$)	Proportion of secondary structure (%)			
	α-Helix	β-Sheet	Turns	Random Coils
0	26	37.5	6.7	29.8
5	27.8	33.8	8.4	30
15	29.1	31.4	9.4	30
25	30.2	29.5	10.3	29.9
35	30.3	26.9	11	31.9

References

[1] Y.-J. Lee, G. Moon, H. Cha, J. Lee, E.E. Kwon, Perfluoroalkyl chain-length-dependent environmental fate and treatment outcomes of PFAS in water, npj Clean Water 9(1) (2026) 35.

[2] T.M.H. Nguyen, J. Bräunig, K. Thompson, J. Thompson, S. Kabiri, D.A. Navarro, R.S. Kookana, C. Grimison, C.M. Barnes, C.P. Higgins, M.J. McLaughlin, J.F. Mueller, Influences of Chemical Properties, Soil Properties, and Solution pH on Soil–Water Partitioning Coefficients of Per- and Polyfluoroalkyl Substances (PFASs), Environmental Science & Technology 54(24) (2020) 15883-15892.

[3] S.W. Karickhoff, D.S. Brown, T.A. Scott, Sorption of hydrophobic pollutants on natural sediments, Water Research 13(3) (1979) 241-248.

[4] F. Hu, J. An, A. Su, B. Wang, Z. Ding, X. Yan, S. Wei, M. Xu, H. Zhang, Green manure plant and exogenous degrading bacteria synergistically promote atrazine removal by enriching indigenous rhizosphere degraders, Journal of Environmental Sciences (2025).

[5] L. Yue, C. Ge, D. Feng, H. Yu, H. Deng, B. Fu, Adsorption–desorption behavior of atrazine on agricultural soils in China, Journal of Environmental Sciences 57 (2017) 180-189.

[6] M. Tahir, A.U. Hassan, S. Maqbool, B. Barber, W.C. Koskinen, X. Peng, D.J. Mulla, Sorption and Leaching Potential of Isoproturon and Atrazine in Low Organic Carbon Soil of Pakistan Under a Wheat-Maize Rotation, Pedosphere 26(5) (2016) 687-698.

[7] L.E. Nakagawa, L.C. Luchini, M.R. Musumeci, M.M. de Andréa, Behaviour of atrazine in brazilian soils under laboratory conditions, Pesquisa Agropecuaria Brasileira 30(4) (1995) 471-476.
